# Removing N-Terminal Sequences in Pre-S1 Domain Enhanced Antibody and B-Cell Responses by an HBV Large Surface Antigen DNA Vaccine

**DOI:** 10.1371/journal.pone.0041573

**Published:** 2012-07-23

**Authors:** Guohong Ge, Shixia Wang, Yaping Han, Chunhua Zhang, Shan Lu, Zuhu Huang

**Affiliations:** 1 Department of Infectious Diseases, The First Affiliated Hospital of Nanjing Medical University, Nanjing, China; 2 China-US Vaccine Research Center, The First Affiliated Hospital of Nanjing Medical University, Nanjing, China; 3 Department of Medicine, University of Massachusetts Medical School, Worcester, Massachusetts, United States of America; Massachusetts General Hospital, United States of America

## Abstract

Although the use of recombinant hepatitis B virus surface (HBsAg) protein vaccine has successfully reduced global hepatitis B infection, there are still a number of vaccine recipients who do not develop detectable antibody responses. Various novel vaccination approaches, including DNA vaccines, have been used to further improve the coverage of vaccine protection. Our previous studies demonstrated that HBsAg-based DNA vaccines could induce both humoral and CMI responses in experimental animal models. However, one form of the the HBsAg antigen, the large S antigen (HBs-L), expressed by DNA vaccine, was not sufficiently immunogenic in eliciting antibody responses. In the current study, we produced a modified large S antigen DNA vaccine, HBs-L(T), which has a truncated N-terminal sequence in the pre-S1 region. Compared to the original HBs-L DNA vaccine, the HBs-L(T) DNA vaccine improved secretion in cultured mammalian cells and generated significantly enhanced HBsAg-specific antibody and B cell responses. Furthermore, this improved HBsL DNA vaccine, along with other HBsAg-expressing DNA vaccines, was able to maintain predominantly Th1 type antibody responses while recombinant HBsAg protein vaccines produced in either yeast or CHO cells elicited mostly Th2 type antibody responses. Our data indicate that HBsAg DNA vaccines with improved immunogenicity offer a useful alternative choice to recombinant protein-based HBV vaccines, particularly for therapeutic purposes against chronic hepatitis infection where immune tolerance led to poor antibody responses to S antigens.

## Introduction

Hepatitis B virus (HBV), a member of the Hepadnavirus family, is the main pathogen for human viral hepatitis; chronic infection can lead to liver cirrhosis and hepatocellular carcinoma [1]. Although the original virus was discovered in the 1960 s [2], HBV infection remains a major global health issue today. According to the World Health Organization (WHO), an estimated two billion people worldwide have been infected with HBV, more than 300 million have chronic infection, and 600,000 people die every year related to the infection. As of 2012, China, as a region with a high prevalence of HBV infection [3], has an estimated 120 million people infected with the disease [4].

The HBV vaccine has proven effective in preventing HBV infection. The hepatitis surface protein antigen (HBsAg) is the target for protective antibody responses for HBV vaccines [5]. The first generation HBV vaccine, used in the 1980 s, included HBsAg prepared from plasma obtained from HBV infected patients [6] but was discontinued due to safety concerns. Subsequently, several recombinant HBsAg vaccines have been successfully developed and used as routine global human vaccinations. Recombinant S proteins [7], [8], [9], [10], produced either in yeast or CHO cells and referred to as second generation HBV vaccines, are the main forms currently used in regular HBV vaccination throughout the world. Although these recombinant S protein vaccines elicit protective antibody responses in more than 80% of the vaccine recipients after the routine three vaccination regimen, a small percentage of vaccinees do not develop detectable antibody responses even with another boost vaccination [11], [12]. Recombinant protein-based vaccines are also not very effective in eliciting T-cell immune responses. T-cell immunity plays a very important role in controlling chronic HBV infection and may even prevent clinical manifestation of HBV infection in the absence of humoral immunity [13]. Therefore, there is a need to develop improved HBV vaccines that are capable of eliciting protective antibody responses among those non-responders to the recombinant S protein-based vaccines and also to elicit efficient T-cell immune responses.

HBsAg is composed of three related co-carboxyl terminal surface proteins created by different translational initiation sites in the viral S gene: the small (HBs-S), middle (HBs-M), and large (HBs-L) proteins. The HBs-S, consisting of 226 amino acid (aa), is a common region of the three HBsAg; HBs-M has an addition of a pre-S2 domain (55 aa) to the N-terminus of HBs-S; and the HBs-L possess another N-terminal addition of a pre-S1 domain (109–118 aa, depending on the viral isolates). Although the HBs-S based recombinant protein vaccination has been very successful, HBsAg mutations capable of escaping diagnostic detection and antibodies elicited by vaccination have been reported. Universal vaccination has actually accelerated wider epitope range of vaccine-resistant mutants [14], [15], [16]. It has been suggested that the inclusion of PreS1 and PreS2 regions in HBV vaccines may be able to induce an immune response to prevent infection by hepatitis B virus escape variants, which may also mimic the immunological stimulus associated with natural infection [17]. Previous studies have reported that the PreS1 and PreS2 domains not only contain both T- and B-cell-specific epitopes but are also involved in hepatocyte receptor binding [18], [19], [20], [21], [22]. Peptide vaccines based on the Pre-S1 sequence induced protective immunity in nonhuman primate models [23], [24], [25] and antibody responses against Pre-S2 were a marker of HBV clearance [25]. Third-generation HBV vaccines containing preS1 and preS2 antigens have been developed with reported excellent immunogenicity in humans, including rapid onset of antibody responses towards the S-protein of the vaccine [26].

Over the past few years, our group has investigated the relative immunogenicity among the three natural forms of HBV S protein. Our results have demonstrated that the DNA vaccine expressing the middle antigen (HBs-M) induced the highest levels of antibody and CMI responses in mice. Our results also showed that the HBs-S DNA vaccine was also highly immunogenic. However, DNA vaccine expressing the large antigen (HBs-L) was not very immunogenic but the mechanism is not clear [27]. Previous studies indicate that the C-terminal half of the preS1 region controls HBs-L secretion by using its N-terminal retention sequence to prevent the secretion of HBs-L protein [28]. In the current study, we hypothesized that removal of the N-terminal sequence in Pre-S1 will increase secretion of the HBs-L and, thus, may improve the immunogenicity of HBs-L DNA vaccines. The results included in the current report support this hypothesis and provide valuable information on how to optimize the design of modified L antigen-based HBV vaccines for improved antibody and B cell responses for both prophylactic and therapeutic applications.

## Results

### 2.1 Constructing HBs-L(T) DNA vaccine expressing the HBsAg large antigen with a deletion of the first 18 amino acids at the N-terminus

**Figure 1:**
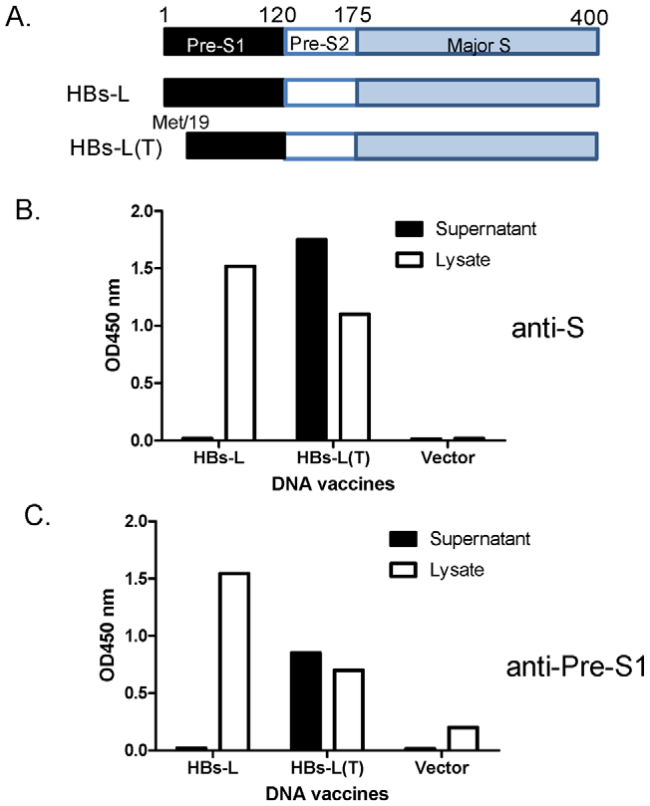
Schematic design of HBs-L DNA vaccines. A. These HBs-L DNA vaccines express the full length large protein of the HBsAg (HBs-L) or the truncated large protein with removal of the first 18 amino acids at the N-terminus, respectively. The amino acid positions for each HBs-L immunogen are as indicated. B and C: The ELISA analyses of the HBs-L and HBs-L(T) protein expressions by the relevant DNA and the empty vector as negative control, using commercially available anti-S (B) or anti-Pre-S mAb (C). “S” and “L” represent the supernatant and lysate of transiently transfected 293T cells.

In our previous study, we found that the DNA vaccine expressing the full length HBsAg large antigen (HBs-L) was not immunogenic in eliciting either antibody or cell-mediated immune (CMI) responses in mice [27]. According to the literature, the N-terminal sequences of HBs-L within the pre-S1 segment prevent the HBs-L protein from being secreted [29], [30], [31] and, therefore, may be responsible for the low immunogenicity of the HBs-L DNA vaccine. In the current study, we designed a modified HBs large antigen DNA vaccine, HBs-L(T), by truncating the first 18 aa in the pre-S1 region (Fig. 1A) to determine if this modified HBs-L antigen DNA vaccine will have improved immunogenicity [32], [33].

Expression of the HBs-L(T) DNA vaccine was examined in transiently transfected 293T cells. Both cell lysate and supernatant collected at 72 hours after transfection were measured by ELISA using monoclonal antibodies that are either specific for the small surface antigen (anti-S, Fig. 1B) or specific for the PreS1 region (anti-PreS1, Fig 1C). While HBs-L antigen was readily detected in cell lysate by both mAbs, basically no HBs-L antigen was detected in the supernatant. Interestingly, transfection with the DNA vaccine HBs-L(T) showed high level expression in supernatant (Fig 1B and 1C) and were detected by both mAbs. The results demonstrated that the removal of the first 18 aa improved secretion of the HBs-L antigen. Further analysis showed that polyclonal animal sera elicited by HBs-M antigen was able to recognize processed surface antigen with lower molecular weight in L(T) transfected 293T cells (Fig. S1).

### 2.2 Comparing the immunogenicity of the full length L (HBs-L) and N-terminal sequence-truncated L (HBs-L(T) DNA vaccines in mice

**Figure 2:**
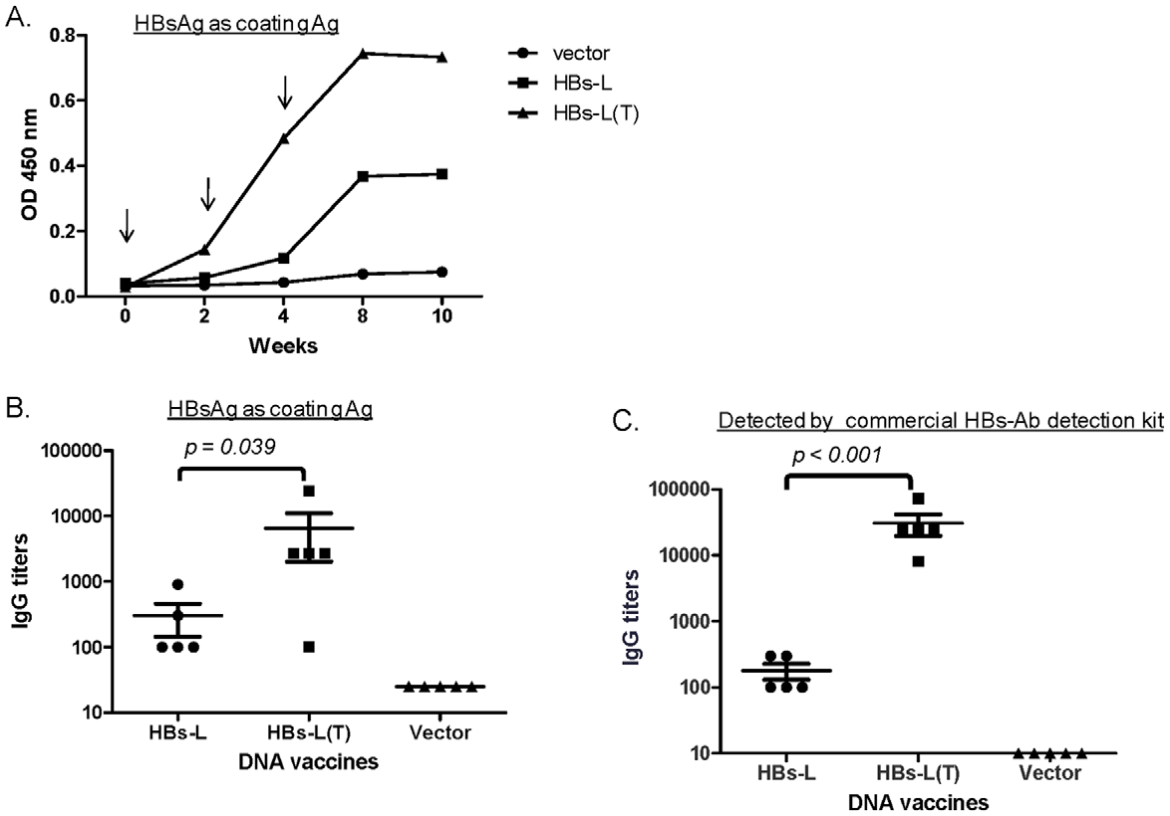
HBs-specific antibody responses in Balb/C mice receiving DNA vaccine expressing HBs-L or HBs-L(T), or the empty vector as negative control. A: Temporal HBs-specific antibody responses in immune mouse sera (1∶500 dilution) detected against commercial HBsAg antigen. The arrows show the time points of DNA immunization. The OD values are expressed as the average of group. B and C: HBs-specific IgG titers (reciprocal serum dilution) induced by HBs-L or HBs-L(T) DNA vaccine or empty vector were detected against commercial HBsAg antigen (B) by ELISA or by a commercial HBs-specific antibody detection kit. Antibody titers were measured using sera collected at 2 weeks after the 3^rd^ DNA immunization. Each symbol represents an individual mouse serum sample. The geometric mean and standard deviation are shown for each group. The statistical differences between each group were determined and groups with p<0.05 are indicated.

The relative immunogenicity of DNA vaccines expressing HBs-L and HBs-L(T) were examined in Balb/C mice by electroporation as previous described [34]. The same amount of DNA vaccine expressing either HBs-L or HBs-L(T) was administered individually to mice at Weeks 0, 2, and 4. Compared to the HBs-L DNA vaccine, the HBs-L(T) DNA vaccine induced HBsAg-specific antibody responses earlier and with higher titers (Fig 2). The mice in the HBs-L-T group produced detectable antibody responses after the first DNA immunizations and titers went up rapidly with each subsequent immunization, while the mice in the HBs-L group started to produce detectable antibody responses against HBsAg after two immunizations (Fig 2A). Peak level antibody titers at two weeks after the 3^rd^ DNA immunization were measured by ELISA against two different types of S antigen preparations: 1) the commercial HBsAg produced from HBV infected patient plasma (Fig 2B), and 2) a commercial HBsAg-specific antibody detection kit (Kehua, Shanghai, China) (Fig 2C). The results demonstrated that the HBs-L(T) elicited significantly higher levels of HBs-specific antibody responses than the HBs-L antigen (p<0.05) (Fig 2B and 2C). While the peak level HBs-specific IgG titers induced by the HBs-L DNA vaccine were very low, the antibody titers elicited by HBs-L(T) reached greater than 1∶10,000. As expected, mice immunized with the empty DNA vector did not show any HBs-specific antibody responses.

**Figure 3:**
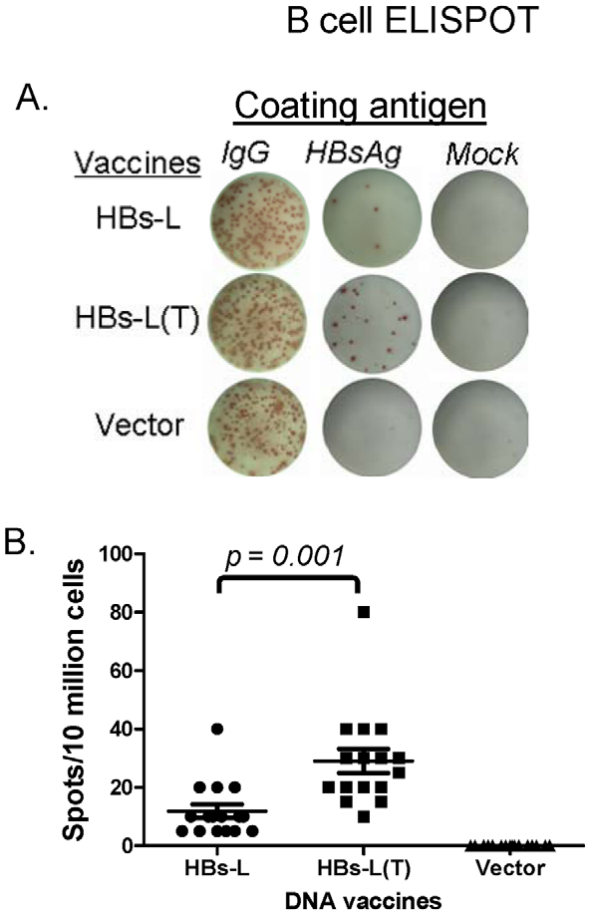
HBs-specific antibody secreting cells (ASC) in splenocytes of immune mice as measured by ELISPOT. Mice were immunized with HBs-L or HBs-L(T) DNA vaccine, or with empty DNA vector as indicated. The coating antigens were anti-mouse IgG, commercial HBsAg and PBS (Mock) for detection of the total IgG, HBsAg-specific or background level ASC in mouse splenocytes. A: Actual sample wells of HBs-specific ASC spots. B: Frequency of HBs-specific ASC per 10 million splenocytes against different HBsAg coating antigens in each group. Data represent the average of spot forming cells (SFCs) per 10 million of splenocytes from each group of mice plus standard error. The splenocytes were collected 1 week after the last (4^th^) DNA immunization. The statistical differences between groups were determined and difference with p<0.05 are indicated. Each symbol represents an individual mouse serum sample. The geometric mean and standard deviation are shown for each group. The statistical differences between each group were determined and groups with p<0.05 are indicated.

HBs-specific antibody-secreting B cell responses were further analyzed to determine whether the above differences in serum antibody responses elicited by HBs-L and HBsL(T) DNA vaccines were the result of a difference at the B cell level. Mouse splenocytes were harvested at 4 weeks after the 3^rd^ DNA immunization and B cell ELISPOT was performed to investigate HBsAg-specific antibody secreting cells (ASC) in immunized mice against the commercial HBsAg (Fig. 3). Consistent with the antibody results, the HBs-L(T) DNA vaccine was much more effective than the HBs-L DNA vaccine in eliciting HBs-specific ASC responses (p<0.05). Total IgG secreting cells captured by anti-IgG antibody were used as positive control and showed no difference among HBs-L, HBs-L(T) and negative control vector groups. The empty DNA vector immunized mice did not produce HBs-specific ASC. Therefore, our ELISPOT results corresponded nicely with the HBsAg antibody response, suggesting that the modified HBs-L(T) antigen is more effective in eliciting HBs-specific B cell responses.

**Figure 4:**
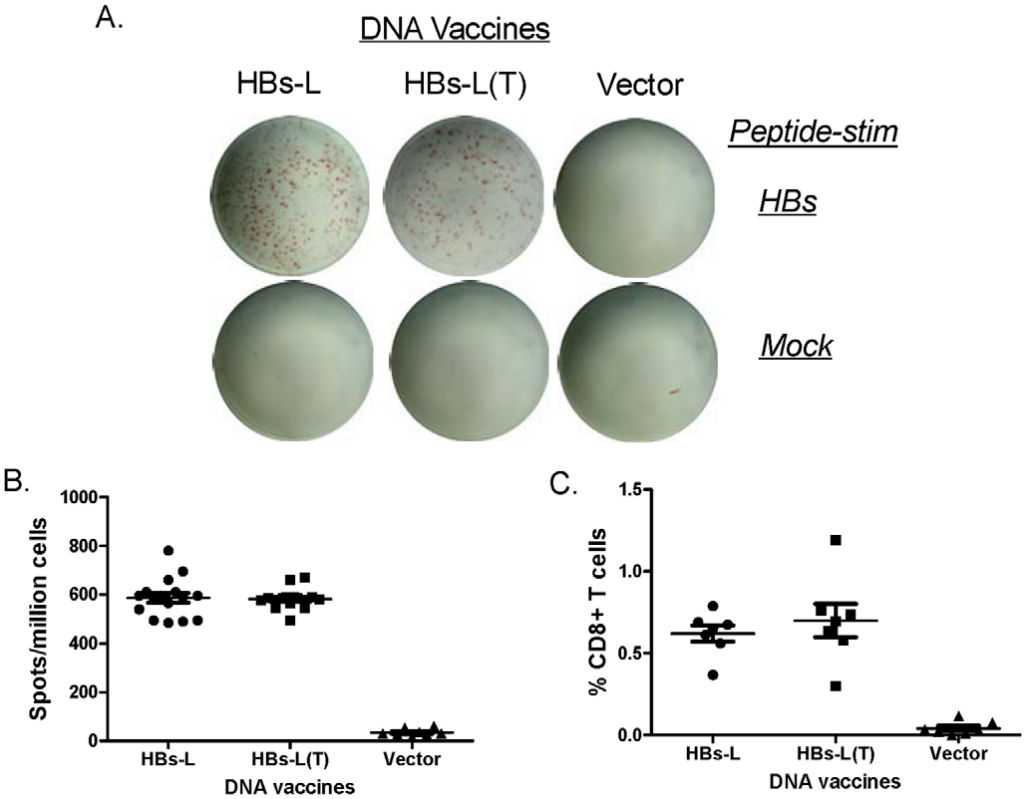
ELISPOT analysis for IFN-γ secretion in mouse splenocytes immunized with HBs-L or HBs-L(T) vaccine or empty vector. (A) Actual sample wells of IFN-γ ELISPOT with mock or HBs peptide stimulation, as indicated. (B) Frequency of HBs peptide-specific IFN-γ spots per million immune mouse splenocytes. (C) The percent HBs-peptide specific CD8+ T cell responses as measured by intracellular staining. The splenocytes were collected at 1 week after the last (4^th^) DNA immunization. Each symbol represents an individual mouse serum sample. The geometric mean and standard deviation are shown for each group. The statistical differences between each group were determined and groups with p<0.05 are indicated.

**Figure 5:**
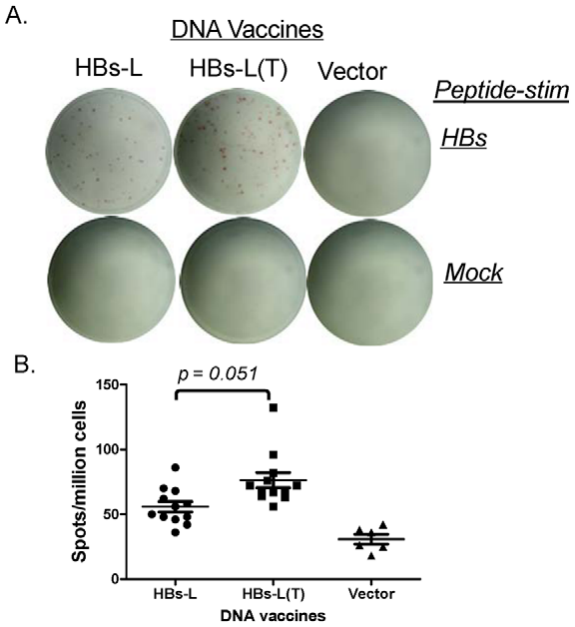
ELISPOT analysis for IL-4 secretion in mouse splenocytes immunized with HBs-L or HBs-L(T) vaccine or empty vector. (A) Actual sample wells of IL-4 ELISPOT with mock or HBs peptide stimulation, as indicated. (B) Frequency of HBs peptide-specific IL-4 spots per million immune mouse splenocytes. The splenocytes were collected at 1 week after the last (4^th^) DNA immunization. Each symbol represents an individual mouse serum sample. The geometric mean and standard deviation are shown for each group. The statistical differences between each group were determined and groups with p<0.05 are indicated.

At the same time, we examined whether there is any difference in T cell responses between HBs-L and HBs-L(T) DNA vaccines. IFN-γ and IL-4 were used in this study, representing Th1 and Th2 T cell immune responses, respectively. ELISPOTs and intracellular cytokine straining studies were conducted with a well-characterized T-cell epitope in S antigen [27]. Total T-cell ELISPOT analysis demonstrated that high levels of HBs-specific IFN-γ responses (an average of 500–600 spots per million splenocytes) were detected in mice immunized with either HBs-L or HBs-L(T) DNA vaccine but there was no difference between these two L antigen DNA vaccines (Fig. 4A and 4B). ICS results further confirmed similar levels of HBs-specific IFN-γ responses in CD8+ T cell populations from both HBs-L and HBs-L(T) DNA vaccine groups (Fig. 4C). ELISPOT results also indicated that the HBs-L(T) immunized mice generated higher levels of IL-4 T-cell responses compared to the HBs-L immunized mice although the difference was not statistically significant (p = 0.051) (Fig 5). In both IFN-γ and IL-4 analyses, mock stimulation with an HIV V3 peptide or splenocytes collected from the empty vector group did not show non-specific T-cell responses (Fig. 4 and Fig 5).

### 2.3 Difference in antibody responses induced either by HBs DNA or by recombinant S protein vaccines

The immunogenicity of HBV DNA vaccines has been well established in small animals including previous studies from our group showing the high immunogenicity of HBs-S and HBs-M antigens [27], [35]. The current report further optimized the HBs-L antigen and demonstrated that DNA vaccines expressing either the large, middle, or small antigen could all induce excellent antibody and cell mediated immune responses. In contrast, the commercially available recombinant HBs protein vaccines mainly induce antibody responses but not CMI. However, there was no direct comparison in one study to show the relative immunogenicity between HBV DNA vaccines and recombinant HBV protein vaccines.

**Figure 6:**
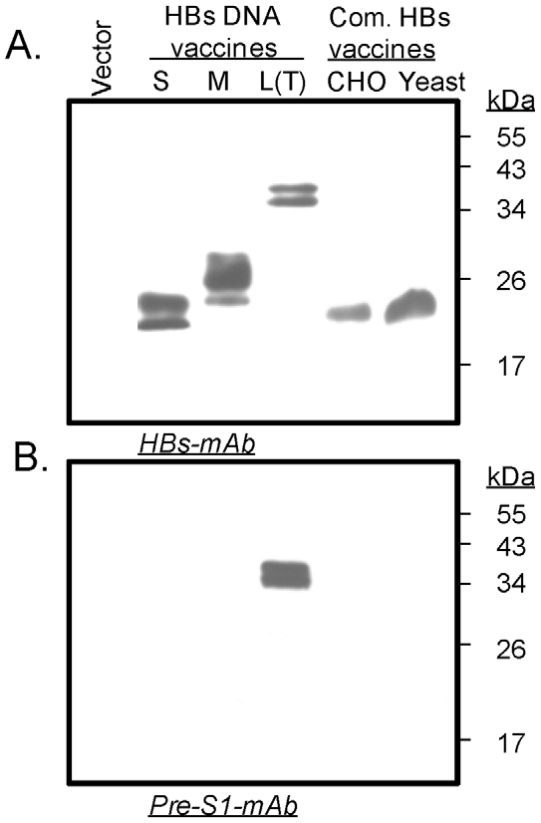
Western blot analyses of HBs-L(T), HBs-M and HBs-S proteins expressed by relevant DNA vaccines in transfected 293T cell lyate and the commercial HBs protein vaccines produced in CHO or Yeast expression system, and the empty vector transformed 293T cell lysate, as indicated. The detection antibodies were anti-S (A) or anti-Pre-S monoclonal antibodies at a concentration of 1 µg/ml.

We next examined the relative antibody responses elicited by different designs of HBs DNA vaccines and compared these responses to those elicited by two HBV protein-based vaccines commonly used in China. One commercially available HBV vaccine in China was produced in CHO cells (CHO-HBs, North China Pharmaceutical Co. Ltd, Shijiazhuang, China) and the other one was produced in a yeast expression system (Yeast-HBs, Abbott, Chicago, IL). The expression of DNA vaccines expressing HBs-L(T), HBs-M, and HBs-S were examined by Western blot using 293T cell samples transiently transfected with these HBsAg DNA vaccines. CHO-HBs and Yeast-HBs vaccines were also included in the analysis. Anti-S mAb was able to recognize HBs-L(T), HBs-M, and HBs-S proteins with expected molecular weights, along with CHO-HBs and Yeast-HBs, which had the expected size of a small antigen (∼27 kD) (Fig 6A). In contrast, anti-PreS1 mAb could only recognize HBs-L(T) because other S antigens lack the PreS1 in their sequences.

Balb/C mice were immunized individually with each of the three DNA and two recombinant protein vaccines at Weeks 0 and 4. For HBs-L(T), HBs-M, or HBs-S DNA vaccines, eletroporation was used to deliver 100 µg DNA vaccines at each immunization while CHO-HBs and Yeast-HBs vaccines were used at 1/10 of human dose by intramuscular needle injection. Immunized mouse sera were collected at 2 weeks after the 2^nd^ immunization and S antigen-specific antibody responses were measured against either the commercial HBsAg produced from HBV infected patient plasma or the HBs-M protein produced by transiently transfected 293T cell supernatant, as previous described.

**Figure 7:**
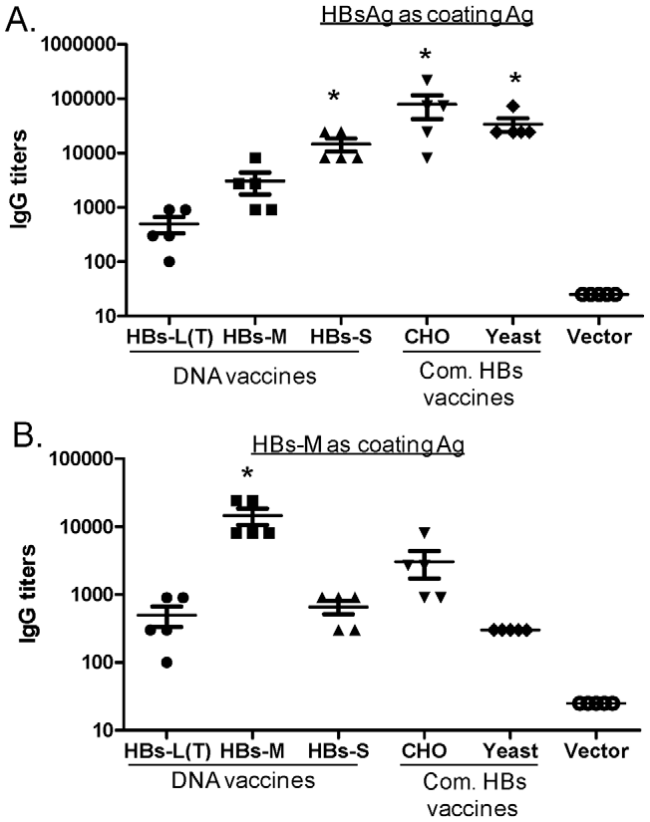
HBs-specific antibody responses in Balb/C mice receiving DNA vaccine expressing HBs-L(T), HBs-M and HBs-S proteins, the commercial HBs protein vaccines produced in CHO or Yeast expression system, or the empty vector as negative control, as indicated. HBs-specific IgG titers (reciprocal serum dilution) induced by various HBs DNA or protein vaccin or empty vector were detected against commercial HBsAg antigen (A) or HBs-M (B) protein expressed by the HBs-M DNA vaccine from transfected 293T cells by ELISA. Antibody titers were measured using sera collected at 2 weeks after the 3^rd^ DNA immunization. Each symbol represents an individual mouse serum sample. The geometric mean and standard deviation are shown for each group. The statistical differences between the individual HBs DNA or protein vaccine groups compared to HBs-L(T) DNA vaccine group were determined and “*” indicate statisticallt significant (p<0.05).

The HBs-S DNA vaccine and two recombinant protein vaccines (CHO-HBs and Yeast-HBs) elicited high level antibody responses against commercial HBsAg (titers >1∶10,000), which were significantly higher than those induced by HBs-L(T) (titers <1,000) or HBs-M DNA vaccines (titers 1∶1000-1∶10,000) (p<0.05) (Fig 7A). Interestingly, antibody specificities of these sera are very different when HBs-M antigen was used (Fig 7B). The HBs-M antigen generated the highest antibody responses against its autologous antigen (titers ∼1∶10,000); CHO-HBs protein vaccine also induced high level HBs-M-specific antibody responses (titers at 1∶1000–1∶10,000). However, antibody titers elicited by the other two DNA vaccines (HBs-L(T) and HBs-S) were much lower. Similarly, Yeast-HBs recombinant protein vaccine also elicited lower antibody responses than CHO-HBs against HBs-M antigen (Fig. 7B). These results may indicate that different forms of HBs vaccines induced antibody responses with preferred antigen conformations and epitopes.

**Figure 8:**
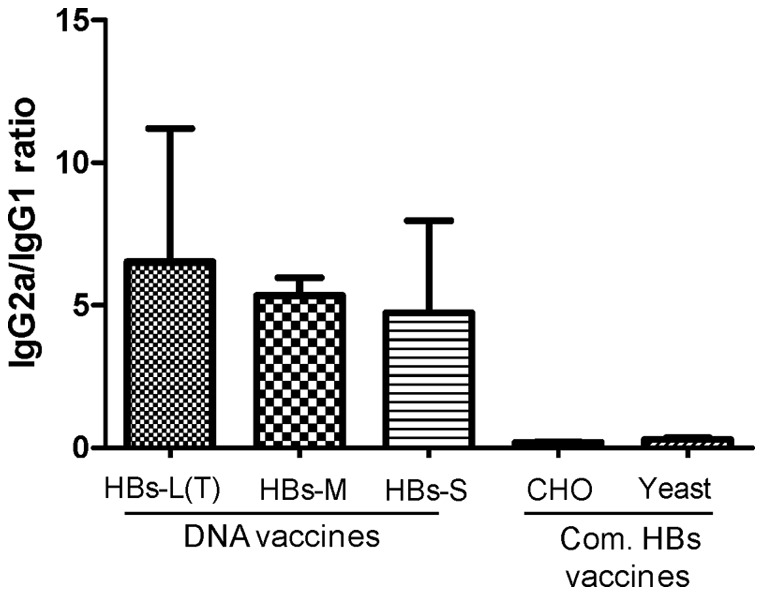
The HBsAg-specific IgG1 and IgG2a responses induced in Balb/C mice receiving DNA vaccine expressing HBs-L(T), HBs-M and HBs-S proteins, the commercial HBs protein vaccines produced in CHO or Yeast expression system, as indicated. The data represent the geometric mean of IgG2a/IgG1 ratios with standard deviations of each group of 5 mice.

The quality of antibody responses elicited by HBs DNA vaccines was further analyzed. The HBsAg-specific IgG1 and IgG2a isotypes in animal immune sera were determined by ELISA (Fig. 8). The recombinant CHO-HBs and Yeast-HBs vaccines induced predominantly IgG1 responses (average IgG2a/IgG1 ratio ∼ 0.2), indicating a strong Th2-biased antibody response. In contrast, three DNA vaccines (HBs-L(T), HBs-M and HBs-S) induced uniformly high level IgG2a responses, supporting a Th1-type antibody response. This comparison provided clear evidence that DNA vaccines are able to elicit predominantly Th1 antibody responses which is not only specific to L(T) antigens.

## Discussion

The small subunit of HBsAg (HBs-S) has been used as the HBV vaccine immunogen for most of the current recombinant protein-based HBV vaccines on the global market. Although, overall, such HBV vaccines are immunogenic, there are “non-responders” to this type of vaccine [3]. They may not have the ability to protect against certain escape mutant viruses including vaccine-resistant mutants and those generated by antiviral treatments [36]. It is now known that the preS1 domain of HBsAg is necessary for attachment and entry of the virus and antibodies to the preS2 domain may inhibit infection both *in vivo* and *in vitro* [37], [38], [39], [40]. Therefore, in order to further enhance the immunogenicity and protective immunity against HBV infection, pre-S1 and pre-S2 domains have been included third generation HBV vaccines.

DNA vaccination is a useful tool to test the immunogenicity of different immunogen designs by antigen engineering. In our previous studies, we investigated the antibody, B cell, and T cell responses in mice receiving DNA vaccines expressing either the large antigen (HBs-L), middle antigen (HBs-M) or small antigen (HBs-S) to compare three forms of HBsAg [27]. Although all three forms of HBs DNA vaccines induced HBs-specific humoral and CMI responses, the HBs-L DNA vaccine was less immunogenic than other two forms, especially in eliciting antibody responses. In the current study, we modified the DNA vaccine expressing the truncated large antigen HBs-L(T) by removing the first 18 amino acids at the N-terminus of the PreS1 domain to make the large antigen more secretable in transfected mammalian cells and to examine whether the modified DNA vaccine HBs-L(T) could induce enhanced HBs-specific antibody and B cell immune responses in mice.

The results demonstrated that the N-terminal truncated HBs-L(T) DNA vaccine could express the antigen in both supernatant and the cell lysate and were detectable by both anti-S or anti-preS1 monoclonal antibodies, while the original HBs-L DNA could only express the antigen in the cell lysate. The truncated large antigen, HBs-L(T), had lower reactivity to the preS1 mAb compared to the wild type HBs-L while both HBs-L and HBs-L(T) had similar reactivity to anti-S mAb, confirming that a deletion in the preS1 region may have affected the epitope recognized by the anti-preS1 mAb. The HBs-L(T) DNA vaccine generated significantly higher levels of HBs-specific antibody responses in mice compared with the wild type HBs-L DNA vaccine, against two different antigens: 1) HBsAg purified from HBV infected patient plasma, and 2) the middle antigen expressed by the HBs-M DNA vaccine. More significantly, HBs-L(T) DNA vaccine elicited higher levels of HBsAg-specific antibody secreting cells, which may account for the higher levels of overall HBsAg-specific antibody responses.

At the same time, the HBs-L(T) DNA vaccine was able to maintain its immunogenicity for eliciting T-cell immune responses as the original HBs-L DNA vaccine. Overall IFN-γ specific T-cell immune responses, as shown by ELISPOT, and IFN-g specific CD8+ T cell responses, as shown by intracellular staining (ICS), were similar in the HBs-L immunized group when compared to levels observed in the HBs-L DNA vaccine group. Slightly higher HBs-specific IL-4 T cell responses were observed in the HBs-L(T) DNA vaccine group when compared with the HBs-L group, however, this difference was not statistically significant.

The above findings imply that the N-terminal sequence-truncated HBs-L(T) antigen enhanced HBs-specific antibody responses and possible Th2 cytokine production by T cells without compromising production of Th1 cytokines, including IFN-γ production from CD8+ T cells. Our results support the use of the HBs-L(T) DNA vaccine as a candidate HBV DNA vaccine since it covers the preS1, preS2 and S antigen regions in one single DNA vaccine.

Once we have established the immunogenicity of HBV large, middle, and small antigens, as tested by DNA vaccination, a side-by-side comparison was conducted with recombinant protein-based HBV vaccines. In the current study, two commercially available recombinant protein HBV vaccines, HBV vaccines expressed by the yeast expression system (Yeast-HBs) and HBV vaccines expressed by the CHO expression system (CHO-HBs), were tested for their immunogenicity in the mouse and compared against the immunogenicity observed for the three forms of HBs DNA vaccines (HBs-L(T), HBs-M and HBs-M).

Although both yeast-HBs and CHO-HBs and different forms of HBs DNA vaccines induced positive HBs-specific antibody responses against either HBsAg purified from HBV patient plasma or HBs-M expressed by DNA vaccine, differences were observed. Antibody responses induced by recombinant protein-based yeast-HBs and CHO-HBs vaccines were predominantly Th2 type antibody responses while the DNA vaccines mainly induced Th1 type antibody responses. Literature has suggested that the Th1 rather than the Th2 antibody responses might be more relevant to protective immunity against viral infections, such as HSV-2 [41]. The findings from the current study provide very useful information for future HBV vaccine design. Specifically, these data are useful for the development of future vaccines for individuals who are resistant to protein-based HBV vaccine and to serve as a novel immune therapeutic approach to people with chronic HBV infection. While traditionally T cell mediated immune responses were considered as a possible mechanism to elicit therapeutic effect, it is also possible to have vaccines that can induce improved antibody and B cell immune responses as shown here with modified L antigen when delivered as a DNA vaccine.

## Materials and Methods

### 4.1 Construction of HBs-L and HBs-L(T) DNA vaccines

The codon optimized large HBsAg (HBs-L) gene coding for the large HBsAg protein, including pre-S1, pre-S2, and S domains, was chemically synthesized by Geneart (Regensburg, Germany), with added restriction enzyme sites of PstI and BamHI for subcloning purposes immediately upstream of the start codon and downstream of the stop codon, respectively [27]. The gene insert for truncated large HBsAg (HBs-L(T))(see fig1 A), including truncated 2–19 amino acids PreS1, pre-S2 and S genes, was PCR-amplified from the L HBs gene template using the following primers: HBV L(T)-Adropt-1 (5′ TACACTCTGCAG_ PstI_ ATGAACCCCCTGGGCTTCTTC 3′) and HBV L(T)-Adr-opt-2 (5′GAGCTCGGATCC_BamHI_TCAGATGTACACCCAC 3′), and then subcloned into pSW3891. Each individual DNA vaccine plasmid was prepared in large amounts from *Escherichia coli* (HB101 strain) with a Mega purification kit (Qiagen, Valencia, CA, USA) for both *in vitro* transfection and *in vivo* animal immunization studies.

### 4.2 In vitro expression of HBs proteins

The expression of the HBs DNA vaccine constructs was examined by transient transfection of 293T cells as previously reported [42]. Transfection was done when cells were at approximately 50–70% confluence on 60-mm dishes by polyethylenimine(PEI) co-precipitation, using 10 µg of plasmid DNA per dish. The supernatants and cell lysates were harvested 72 hours after transfection. The amount of HBsAg produced was determined by standard HBsAg (HBs-S) and PreS1 detection EIA kits from Shanghai Kehua Bio, Inc (Shanghai, China).

### 4.3 Western blot analysis

The S antigens produced in transiently transfected 293T cells were analyzed by Western blot. Samples were subjected to denaturing SDS-PAGE and blotted onto PVDF membrane (BioRad). Blocking was done with 5% non-fat dry milk with 0.1% Tween-20 in PBS. Commercial anti-HBs-S mouse monoclonal antibody 1023 and anti-PreS1 monoclonal antibody AP1 from Santa Cruz Biotechnology, Inc. were used as detecting antibodies at 1∶500 dilution. The membranes were washed with blocking buffer and reacted with AP-conjugated goat anti-rabbit (Tropix) at a 1∶5000 dilution [35], [43]. After final wash, Western light substrate was applied to the membranes for 5 minutes. Once the membranes were dry, Kodak films were exposed to the membrane and developed with an X-Omat processor.

### 4.4 Immunization of mice

BALB/c mice (6–8 weeks old), purchased from Shanghai Animal Center, Chinese Academy of Science, were used for immunogenicity studies. The mice were housed in the Department of Animal Medicine at the Nanjing Medical University in accordance with approved protocol. Animals were sedated with Ketamine (0.1 mg/g of body weight) for DNA immunizations. Electroporation was used for DNA vaccine delivery as previous described [34]. An electroporator (SCIENTZ-2C) manufactured by Scientz Co., Ltd (Ningbo, China) was used. Briefly, following intramuscular injection (IM) of DNA vaccine or vector control (100 μg per mouse), the injection sites were electroporated using the following parameters: 50 V, 30 ms and 30 Hz. The DNA plasmid was delivered at two different sites in the quadriceps muscle for each immunization. A total of three immunizations were given at Weeks 0, 2, and 4. Serum samples were taken prior to the first immunization and two weeks after each immunization to measure HBs-specific antibody responses. Mouse splenocytes were collected at 1 week after the last boost for HBs-specific T and B cell assays. The final splenocyte preparations were 5×10^6^ cells/ml in R10 medium (RPMI1640 medium with 10% FBS, 1% Penicillin-Streptomycin).

### 4.5 Ethics statement

This study was carried out in strict accordance with the recommendations in the Guide for the Care and Use of Laboratory Animals of the National Institutes of Health, USA. The protocol was approved by the review of Institutional Animal Use Committee at Nanjing Medical University, China.

### 4.6 Enzyme-linked immunosorbent assay (ELISA) for serum samples

An ELISA was conducted to measure HBs-specific antibody responses in immunized mice, as previously described [27]. Levels of isotypes IgG antibodies in mouse serum were determined. The 96-well flat-bottom plates were coated with 100 μl (∼ 0.1 µg) of one of the following individual HBs antigens: HBsAg was isolated from HBV infected patient plasma purchased from USBiological (Swampscott, MA, USA), M HBs protein was produced from transiently transfected 293T cell supernatant. Animal sera were incubated for 1 hour at room temperature and washed 5 times with PBS containing 0.1% Triton X-100. The plates were then blocked with 200 μl/well of blocking buffer (5% non-fat dry milk, 4% Whey, 0.5% Tween-20 in PBS at pH 7.2) for 1 hour. After five washes, 100 μl of serially diluted mouse serum was added in duplicate wells and incubated for 1 hour. After another set of washes, the plates were incubated for 1 hour at 37°C with 100 μl of biotinylated anti-mouse IgG (USBiological, Swamscott, MA, USA) diluted at 1∶1000 in Whey dilution buffer (4% Whey, 0.5% Tween-20 in PBS) or anti-mouse IgG1or IgG2a (Sothern BioTech, Birmingham, AL) diluted at 1∶1000 of Whey buffer (4% Whey, 0.5% Tween-20 in PBS). Then, 100 μl of horseradish peroxidase-conjugated streptavidin (Vector Laboratories, Burlingame, CA, USA) diluted at 1∶2000 in Whey buffer was added to each well and incubated for 1 hour. After the final wash, the plates were developed with 3,3′,5,5′ Tetramethylbenzidine (TMB) solution at 100 μl per well (Sigma, St. Louis, MO, USA) for 3.5 minutes. The reactions were stopped by adding 25 μl of 2 M H_2_SO_4_, and the plates were read at an OD of 450 nm. The end titration titer was determined as the highest serum dilution that has an OD reading above twice of that from the negative control serum.

### 4.7 Assays for HBs-specific antibody secreting cells (ASCs)

HBs-specific antibody secreting cells (ASCs) in immune mouse splenocytes were detected, as previous described [27]. Multiscreen Immobilon P membrane White Sterile 96-well plates (IP plates, from Millipore, Billerica, MA, USA) were coated with 100 µl (∼ 0.5 µg)/well of HBsAg or M HBs proteins produced from transiently transfected 293T cells and incubated at 4°C overnight. Then the plates were blocked by the addition of 200 µl of the blocking buffer in each well for 2 h at 37°C. Freshly isolated splenocytes (100 µl/well, 2 million cells/well) in R10 medium with 0.1% β-ME were incubated in duplicate wells for 4 h at 37°C. The plates were then washed, incubated with 100 µl of biotinylated goat-anti- mouse IgG diluted at 1∶1000 in PBST (0.05% Tween-20 in PBS at pH 7.2) with 1% fetal bovine serum (FBS) at 37°C for 2 hours. After additional washes, 100 µl of HRP-conjugated Streptavidin complex diluted at 1∶2000 in PBST with 1% FBS was added to each well and incubated at 37°C for 1 h, then spots were developed by a 35-min color reaction using AEC coloring systems. The number of HBsAg-specific ASCs was counted and calculated.

### 4.8 Analysis of HBsAg-specific T cell responses by enzyme-linked immunospot (ELISPOT) assay

Gamma interferon (IFN-γ) and interleukin 4 (IL-4) ELISPOT assays were performed to detect the HBs peptide-specific T cell responses in mouse splenocytes, as previously described [27], [35]. Mouse IFN-γ kit (U-CyTech Biosciences, Netherlands) and IL-4 ELISPOT kit (U-CyTech Biosciences, Netherlands) were used to detect HBs-specific IFN-γ and IL-4 T cell responses, according to manufacturers' directions. Briefly, the IP-plates were coated with 5 μg/ml of purified rat anti-mouse IFN-γ or rat anti-mouse IL-4 in PBS at 4°C overnight. After the plates were washed three times with PBS, each plate was blocked by the addition of 200 µl of the blocking buffer in each well for 2 h at 37°C. The HBs relevant peptide used was a CD8 + cell-restricted HBs peptide (IPQSLDSWWTSL) [35] and the non-relevant peptide was the HIV Env V3 (IGPGRAFYT) peptide, which served as the negative control. The peptides (final concentration 5 μg/ml) were added to the wells with 100 μl of freshly isolated splenocytes (200,000 cells/well in R10 medium) in duplicate. The plates were incubated at 37°C with 5% CO2 for 24 hours. The plates were then washed, incubated with 100 µl of biotinylated rat-anti-mouse IFN-γ or rat-anti-mouse IL-4, respectively (1 µg/ml in dilution buffer from the kit), and incubated at 37°C for 1 hour. After additional washes, 100 µl of HRP-conjugated Streptavidin complex was added to each well in above dilution buffer at 37°C for 1 hour. The plates were washed, and spots representing individual IFN-γ or IL-4 producing cells were detected after a 35-min color reaction using AEC coloring system. The spot-forming cells (SFCs) were counted and the results were expressed as the number of SFCs per million input cells. The number of peptide-specific IFN-γ-secreting T cells was calculated by subtracting the background (no-peptide) control value from the established SFCs count.

### 4.9 Intracellular cytokine staining (ICS) for HBsAg specific IFN-γ production assay

The freshly isolated splenocytes (106 cells in 200 µl) were cultured at 37°C for 5 h in 96-well round-bottom plates in completed R10 medium supplemented with 0.5 mg/ml Brefelding A(Sigma, St. Louis, MO, USA). Stimulatory conditions included 5 µg/ml of the given HBs peptide or 4 µg/ml of HBsAg, as described above [35], and a unrelated HIV Env V3 peptide was used as the negative control. After incubation, the following procedures were performed at 4°C: first, cells were washed with FACS buffer (PBS with 2% FBS and 0.01% sodium azide) and then incubated for 10 min in 100 µl of FcBlock (2.4 G2 MAb, BD Biosciences); after washes, the CD8- APC-conjugated anti-CD8 mAb (clone 53–6.7; BD Biosciences) and cy5.5-conjugated anti-CD3 mAb (clone 145-2C11; BD Biosciences) were used for the cell surface staining for 30 min at 4°C and then washed. Being subjected to intracellular cytokine staining, the cells were fixed and permeabilized using the Cytofix/Cytoperm kit in accordance with the manufacturer's recommendations (BD Pharmingen). The cells were then stained using FITC-conjugated rat anti-mouse IFN-γ MAb (clone XMG1.2) for 30 min at 4°C. Flow cytometry analysis was performed using a FACScalibur (Becton–Dickinson) and data were analyzed using FlowJo software (TreeStar, Inc., Ohio).

### 4.10 Statistical analyses

Student's t-test was used to analyze the differences in antibody, ASC, ICS, and T-cell responses between different animal immunization groups.

## Supporting Information

Figure S1 **Western blot analysis of HBs-L(T) expression using various anti-S antibodies.** A: anti-HBs-M mouse sera; B: anti-HBs mAb; and C: anti-Pre-S1 mAb, respectively. Transfected 293T cell lysates of HBs-L(T) DNA vaccine or empty vector were loaded as indicated.
(TIF)
