# Supplementary figures and images for: Removing N-Terminal Sequences in Pre-S1 Domain Enhanced Antibody and B-Cell Responses by an HBV Large Surface Antigen DNA Vaccine
Source: PLoS One. 2012 Jul 23;7(7):e41573. doi: 10.1371/journal.pone.0041573 (PMC3402421; doi:10.1371/journal.pone.0041573)

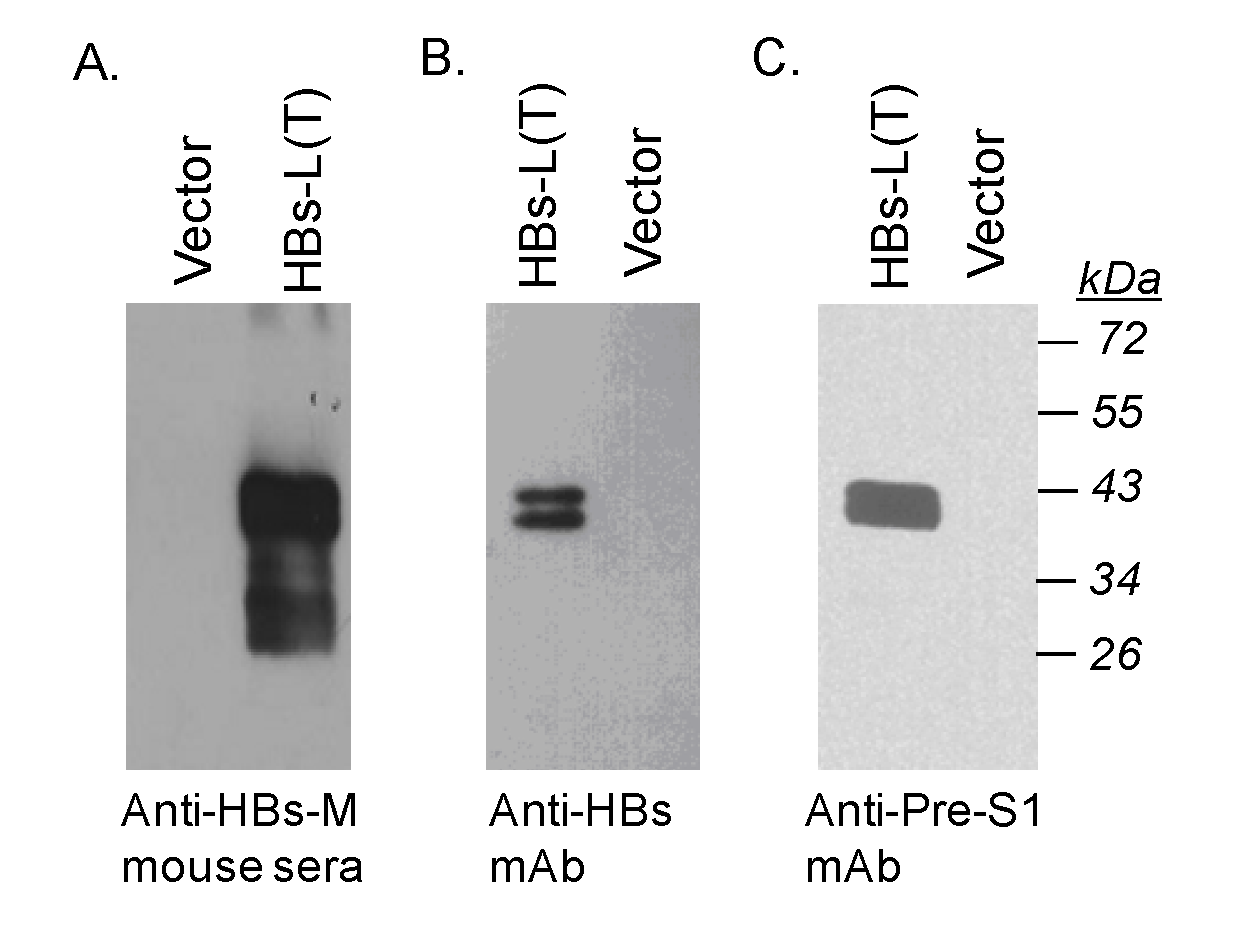

Supplement: Figure S1 — Western blot analysis of HBs-L(T) expression using various anti-S antibodies. A: anti-HBs-M mouse sera; B: anti-HBs mAb; and C: anti-Pre-S1 mAb, respectively. Transfected 293T cell lysates of HBs-L(T) DNA vaccine or empty vector were loaded as indicated. (TIF) [file pone.0041573.s001.tif]
